# Accumulation of Silicon and Changes in Water Balance under Drought Stress in *Brassica napus* var. *napus* L.

**DOI:** 10.3390/plants10020280

**Published:** 2021-02-01

**Authors:** Diana Saja-Garbarz, Agnieszka Ostrowska, Katarzyna Kaczanowska, Franciszek Janowiak

**Affiliations:** The Franciszek Górski Institute of Plant Physiology, Polish Academy of Sciences, Niezapominajek 21, 30-239 Kraków, Poland; a.ostrowska@ifr-pan.edu.pl (A.O.); kkaczanowska87@gmail.com (K.K.); f.janowiak@ifr-pan.edu.pl (F.J.)

**Keywords:** orthosilicic acid, iron, canola, water management, water deficit, stress tolerance

## Abstract

The aim of this study was to investigate the accumulation of silicon in oilseed rape and to characterize the changes in chosen water balance parameters in response to drought. The following parameters were estimated: water content, osmotic and water potential, evapotranspiration, stomatal conductance and abscisic acid level under optimal and drought conditions. It was shown that oilseed rape plants accumulate silicon after its supplementation to the soil, both in the case of silicon alone and silicon together with iron. It was revealed that silicon (without iron) helps maintain constant water content under optimal conditions. While no silicon influence on osmotic regulation was observed, a transpiration decrease was detected under optimal conditions after silicon application. Under drought, a reduction in stomatal conductance was observed, but it was similar for all plants. The decrease in leaf water content under drought was accompanied by a significant increase in abscisic acid content in leaves of control plants and those treated with silicon together with iron. To sum up, under certain conditions, silicon is accumulated even in non-accumulator species, such as oilseed rape, and presumably improves water uptake under drought stress.

## 1. Introduction

Plant water economy is associated with the processes of uptake, transport, use and loss of water, and its balance depends on the impact of abiotic and biotic environmental factors. The majority of environmental stress factors have a direct impact on the disturbance of water balance in the plant and stimulate a range of complex cellular and physiological responses which initiate the implementation of water-saving strategies [1]. One of the most important factors affecting plant–water relations is soil drought. Drought-related stress hinders plant production, and as arid and semi-arid regions already constitute over 30% of the world’s land surface, it is a serious threat to agriculture [2]. This effect is further exacerbated by climate change caused by human activity [3]. Plants subjected to drought undergo morphological as well as physiological and biochemical changes [4,5].

Oilseed rape (*Brassica napus* var. *napus* L.) is a plant species widely cultivated in Poland and worldwide, but its proper growth and subsequent crop are significantly regulated by the availability of water in the environment. As has been demonstrated thus far, *Brassica napus* is considered a species exhibiting a significant degree of osmotic adjustment to prolonged drought conditions compared to other Brassica species [6] and the cultivar Markus used in the study is one of three best yielding population cultivars of spring oilseed rape in Poland, with high-fat, low-glucosinolate content, and high yield potential of 101% of the model cultivar for oilseed rape [7]. This is particularly important also because the cultivation of oilseed rape throughout the world is changing very dynamically due to adverse environmental conditions associated with the occurrence of drought. As demonstrated by the United States Department of Agriculture, in 2018, over 70 million tons of oilseed rape were harvested worldwide, which placed oilseed rape second after soybean in terms of total global oil plant production. Due to drought in 2019/2020, the yield dropped to about 62 million tons [8].

An increasingly important role in the regulation of plant resistance to drought stress is attributed to silicon (Si), but its effectiveness might vary depending on soil water conditions during the vegetation period [9]. It plays a protective role against the adverse effects of drought by improving plant hydration, but also enhances water use efficiency in many species [2,10,11,12,13,14,15]. As reported by Oskabe et al. [16], plants maintain a higher water level through osmotic adjustment under drought conditions. However, in many species, a decrease in leaf water content and water potential is observed as a result of drought [17,18], with the use of Si significantly increasing the values of these parameters [19,20]. In rice, Si supplementation under drought caused an increase in water content and osmotic potential in roots and leaves, as well as an increase in turgor [20]. It was shown that in *Poa pratensis* L. under drought, water content in leaves after silicon supplementation increased by 33% [21], with a similar response observed in the case of maize [22]. However, osmotic adjustment is not always associated with increased values of the studied parameters. As reported by Sonobe and Hattori et al. [23], in sorghum after the application of silicon, there were no changes in water content, but there was a decrease in root osmotic potential, which may be due to the accumulation of soluble sugars and amino acids such as alanine or glutamic acid. The increase in leaf water potential may also result from Si deposition in leaves, causing a reduction in transpiration [24,25,26]. Silicon makes up about 29% of the earth’s crust [27], but it occurs mainly as insoluble crystalline aluminosilicates, which are inaccessible to plants [28]. Therefore, it is not universally recognized as an element indispensable for plants, which are able to carry out their lifecycles without it [29]. Nevertheless, Si is considered to be “quasi-essential” [30] because of its many benefits. Due to the growing interest in the positive effects of silicon on plants, as well as its high effectiveness arising from its application method [31,32], Si preparations acting as plant fertilizers [33], which effectively improve yield quantity and quality [29], have started to appear on the market. Currently, research on the role of silicon in plants is conducted on many levels [34], including in the context of drought [15]. Recently, much attention has been paid to how its role is stimulated or inhibited by the presence of other elements, including iron [35,36], due to its significance in plant development [37]. However, determining the role of Si in plant response to water shortage in the soil is very difficult. Mechanistic understanding of its role in abiotic stress resistance is relatively limited [29], but new studies are constantly being published on this subject [15]. It is expected that the use of silicon as a fertilizer to increase drought resistance of plants will become a growing agricultural trend in the near future [38]. Depending on the species, divergent strategies are observed because plants balance the rate of water uptake and loss at the leaf level [34,39]. 

As plants of the *Brassicaceae* family are not widely recognized as good silicon accumulators, no in-depth research has been conducted thus far on how silicon supplementation of these plants can affect its accumulation and improvement of water management parameters. The validity of such studies is confirmed by the results of Sonah and Deshmukh et al. [40], suggesting that oilseed rape may have proteins other than typical Si transporters (such as Lsi1 in rice or maize), which are responsible for transporting this element, and thus influence its accumulation in the plant. Moreover, due to the fact that oilseed rape is, on the one hand, extremely sensitive to temporary soil water shortage, and on the other, an important crop from the agricultural point of view, commercial silicon preparations stimulating plant growth are very often recommended for the purpose of protection against drought, also in the case of oilseed rape.

In our study, we formulated a hypothesis that oilseed rape, although currently classified as a non-accumulator species, does accumulate Si under the conditions of its high concentration in the soil, and this accumulation of Si in the above-ground plant part affects the functioning of the plant, especially in the conditions of water deficit. The purpose of the study was to verify this hypothesis, firstly by analyzing total Si accumulation in the above-ground parts of oilseed rape plants after Si supplementation. We decided to use a higher concentration of Si than that proposed by Sonah and Deshmukh et al. [40], calculated on the basis of its concentration in the recommended dosage of the commercial preparation *Optysil*. Next, the physiological basis of water balance regulation by silicon in response to drought was investigated, by analyzing water content, osmotic and water potential, evapotranspiration, stomatal conductance of leaves and abscisic acid (ABA) level under optimal and drought conditions.

## 2. Results

Firstly, the analysis of Si in plants growing in optimal conditions showed that Si supplementation significantly changed its content in oilseed rape plants. The analysis of total Si content in the above-ground part of plants growing under optimal conditions revealed that significantly higher Si content compared to control was found in the shoots supplemented with silicon, and the highest in those treated with silicon together with iron (Table 1).

The analysis of water relations parameters of oilseed rape plants growing under the conditions of optimal soil water content showed that significant differences between plants were usually visible one week after the third application of solutions containing silicon (Table 2).

It was shown that plants watered with Si and Si + Fe in optimal growth conditions lost water faster than control plants one week after the second and third watering (Table 2). The response of the plants was the same regardless of whether they were treated with Si or with Si + Fe. The youngest plants with the shortest exposure to silicon—one week after the first watering—watered with Si + Fe or only Si, were observed to maintain a lower value of osmotic potential compared to control plants. At later stages of plants’ growth and with greater availability of Si in the soil, these differences compared to control were reduced, and additionally, one week after the third watering, a significant increase in this parameter was observed in plants of all the tested groups. 

The analysis of Si in plants growing under drought showed that the greatest amount of silicon in the shoot was found in plants watered with Si + Fe, while the greatest amount of silicon in the leaf blade and petiole was found in plants watered only with silicon solution (Table 3).

**Figure 1:**
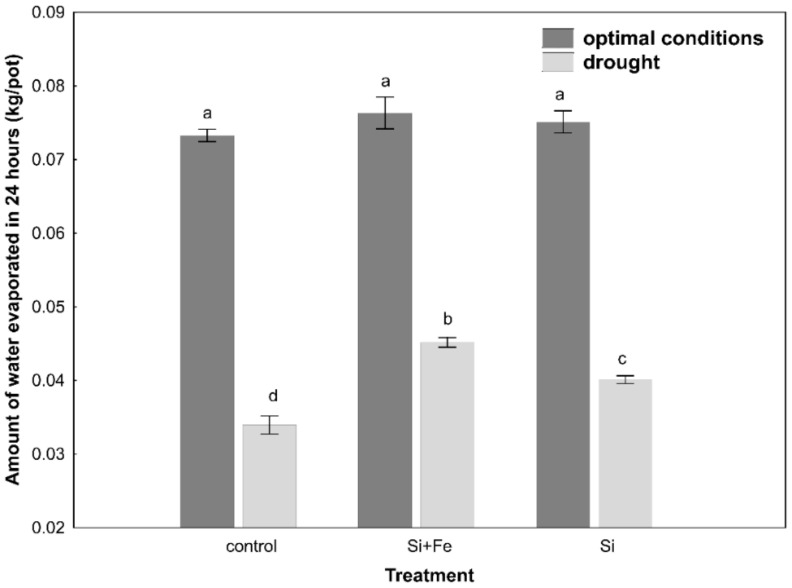
Amount of water evaporated in 24 h. Measurements were performed on pots with oilseed rape plants every day for 10 days during growth under optimal conditions or drought (Experiment 2). Prior to the 10 days, the plants grew for 44 days under optimal conditions and were watered three times with water (control), silicon and iron (Si + Fe), or only silicon (Si). The means (n = 15) ± SD (standard deviation) marked with the same letter do not differ significantly (Fisher’s multiple range test, *p* < 0.05).

Limiting water availability in the soil significantly affected the regulation of water balance in *Brassica napus* var. *napus* L., which was differentiated depending on the presence of silicon. It was shown that under optimal growth conditions, the amount of evapotranspirated water was significantly higher than under drought conditions, and while the application of Si had no significant effect on evapotranspiration under optimal conditions, there was a significant increase in evapotranspiration in the presence of silicon under drought (Figure 1).

**Figure 2:**
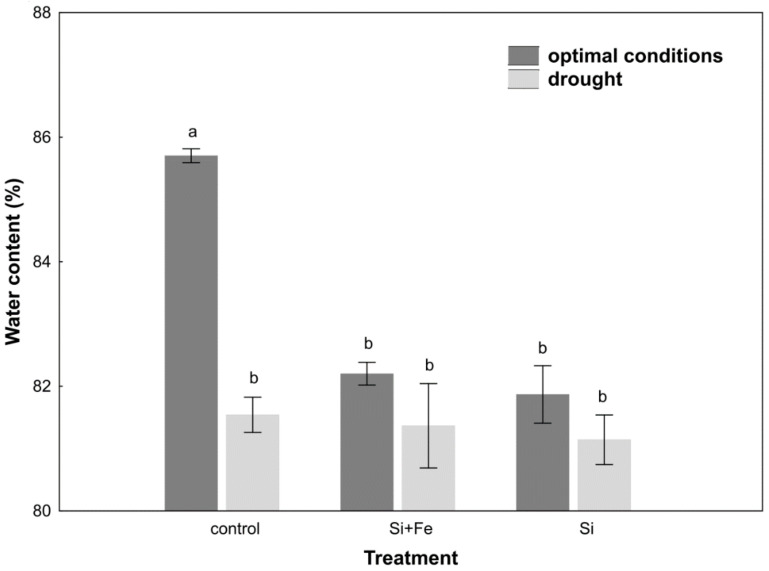
Water content in leaves of oilseed rape. Plants grew first for 44 days under optimal conditions and were watered three times with water (control), silicon and iron (Si + Fe), or only silicon (Si), and afterwards for 10 days under optimal conditions or drought, after which water content measurements were performed (Experiment 2). The means (n = 9) ± SD marked with the same letter do not differ significantly (Duncan’s multiple range test, *p* < 0.05).

The water content measurements performed for oilseed rape plants growing under optimal and drought conditions showed that silicon (Si) and silicon with iron (Si + Fe) significantly reduced leaf water content of plants growing under optimal conditions compared to control plants (Figure 2). 

In control plants, significant water content differences were observed between plants growing under optimal conditions and drought. In plants growing under stress conditions, water content was comparable in all studied groups. The response of the plants watered with Si and Si + Fe under drought was the same as under optimal conditions (Figure 2). In addition, a significant decrease was observed in the value of osmotic and water potential in all plants growing under drought compared to control ones (Table 4).

Under optimal and drought conditions, the osmotic potential of plants supplemented only with Si was lower than in plants treated with Si + Fe.

**Figure 3:**
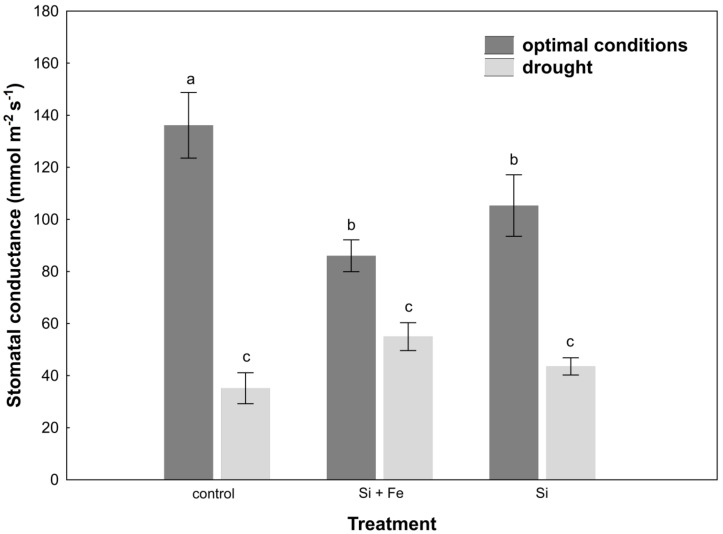
Stomatal conductance in leaves of oilseed rape. Plants grew first for 44 days under optimal conditions and were watered three times with water (control), silicon and iron (Si + Fe), or only silicon (Si), and afterwards for 10 days under optimal conditions or drought, after which stomatal conductance measurements were performed (Experiment 2). The means (n = 9) ± SD marked with the same letter do not differ significantly (Duncan’s multiple range test, *p* < 0.05).

The measurement of stomatal conductance of leaves under optimal growth conditions revealed the highest conductance in plants not supplemented with silicon (Figure 3).

**Figure 4:**
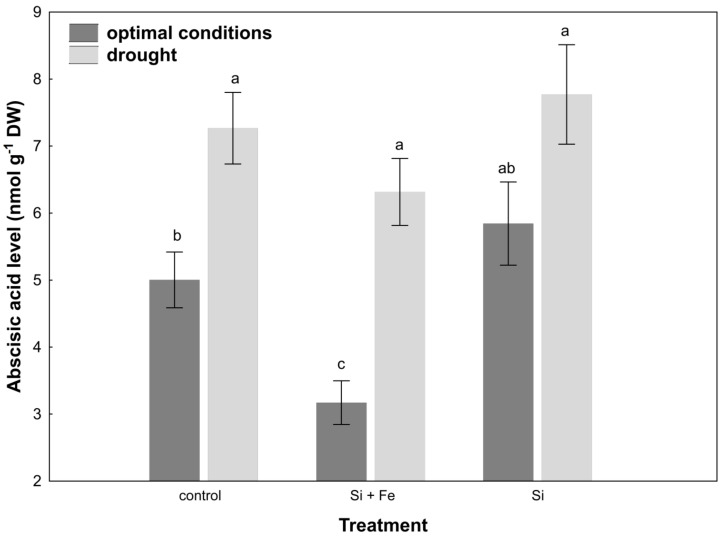
Abscisic acid (ABA) in leaves of oilseed rape. Plants grew first for 44 days under optimal conditions and were watered three times with water (control), silicon and iron (Si + Fe), or only silicon (Si), and afterwards for 10 days under optimal conditions or drought, after which ABA measurements were performed (Experiment 2). The means (n = 9) ± SD marked with the same letter do not differ significantly (Duncan’s multiple range test, *p* < 0.05).

Under water-deficit conditions, a significant reduction in stomatal conductance was observed in all plants, but this change was not dependent on the presence of silicon. Drought stress increased abscisic acid (ABA) content in the leaves of control plants and plants watered with Si + Fe compared to those growing under optimal conditions (Figure 4).

## 3. Discussion

The availability of water in the environment influences its uptake by plants. Water-deficit in the plant is mainly caused by the limitation of its occurrence in the soil (soil drought) or by the existence of obstacles in its uptake from the environment (physiological drought), which in both cases causes a disturbance in plant water balance. The negative effects of these processes are mitigated by supplementing plants with silicon. Despite oilseed rape’s poor ability to accumulate silicon, in this work, it was shown that it is accumulated in the above-ground plant part to a greater extent compared to control, both under optimal growth conditions and under drought, which may affect its protective role in the process of plant growth and development (Table 1 and Table 3). Moreover, it was observed that both under optimal conditions (Table 1) and under drought (Table 3), a significantly higher silicon content was found in the petioles compared to the leaf blades. Whereas there was no linear correlation between Si content and physiological parameters. This effect requires more detailed analyses of the location of Si deposition in the plant, but it seems to be important for the hydraulic conductivity of the petioles, affecting plant water management, which will be the subject of further research.

In oilseed rape leaves under optimal soil water conditions, silicon was shown to cause a decrease in water content compared to control plants, which might suggest their faster senescence (Table 2). At this stage of research, however, it is difficult to conclude about the mechanism of these changes. At the same time, it was shown, similar to the studies by Habibi [36], that water content of oilseed rape leaves decreased under drought stress, though in our studies it was not confirmed that silicon helps maintain relatively higher water content in the plant under drought conditions (Figure 2). However, it was observed that Si ameliorated the effects of water scarcity, stabilizing leaf water content in plants under drought. The analysis of changes in the osmotic potential in optimal conditions does not indicate the accumulation of osmotically active substances (Table 2). It can be assumed that this effect results from improved water uptake and transport through the roots, as well as higher hydraulic conductivity of the root [31], which will be analyzed in subsequent studies. In drought, there was no significant reduction in the osmotic potential in the leaves (Table 4). The main reason might be the frequently emphasized huge difference in osmotic adjustment between plants [31]. For example, in sorghum, it has been observed that the enrichment of the soil with silicon during drought stress increases the absorption of water by the plant and helps maintain its high content in leaves [37], but it is not a rule and even within one Brassica genus there may be differences [6]. On the other hand, some well-known effects were observed, connected with osmotic adjustment of plants to drought stress through a decrease in water potential, as in the case of the study by Morgan [41]. However, it was found that water content in oilseed rape leaves was definitely not associated with maintaining greater water content in the soil, because the amount of water lost due to evapotranspiration within 24 h was the same for all plants under optimal growth conditions (Figure 1). Under drought conditions, on the other hand, it was shown that the greatest amount of water evaporated over 24 h from plants and soil treated with silicon (Figure 1). In view of the fact that under drought, silicon-supplemented plants had a significantly higher Si content in their stems than control plants (Table 3), it can be once again suggested that in this particular case, the plant’s strategy regarding the use of Si has more to do with its positive effect on water uptake than with limiting water loss. The strategy of limiting water loss, which can potentially be regulated by the presence of silicon, was analyzed on the basis of leaf stomata conductance determining changes in transpiration. However, there are contradictory reports in the literature on the impact of Si on the transpiration index. According to previous studies, the application of silicon can significantly increase the transpiration rate in plants subjected to drought, e.g., wheat [39] and sorghum [40,41], alleviate the effects of drought stress by reducing the transpiration rate [7,42], e.g., in maize [43,44], or not play any significant role, e.g., in cucumber [41]. When plants begin to sense drought stress, they start reducing the loss of water from leaves mainly through stomata closure, which was also confirmed in this study.

In the case of oilseed rape, it was observed that under optimal soil water conditions, transpiration did actually decrease after the application of silicon (Figure 3), i.e., Si had a significant stimulating effect also under optimal conditions. This effect was also demonstrated by Agarie and Uchida et al. [42] in the case of rice. It also disproves the hypothesis that silicon effects manifest only when plants grow under abiotic and biotic stresses, reported by Gao and Cai et al. [43]. Moreover, by partially closing the stomata under optimal growth conditions, silicon can significantly increase water use efficiency, which in the case of arable crops in the conditions of increasing freshwater prices, is of great importance for the agriculture of the future. Under drought, a reduction in stomatal conductance was generally observed, but it was similar for all plants within treatment. Si was not shown to play a protective role in this aspect. Similar results were obtained by Habibi [44], who showed that in the studied oilseed rape cultivar, stomatal conductance was comparable under drought in plants supplemented and not supplemented with silicon. It might be explained by the fact that the level of initiated drought was so high (30% of field water capacity, FWC) that stomata closure was the final defense of the plant against evapotranspiration, regardless of the presence of Si in the environment. It is even more probable given that in nature, opposite situations occur where transpiration from leaves under drought is amplified by the presence of silicon, e.g., in rice [20]. The present study also showed that the decrease in leaf water content under drought stress significantly increased ABA content in leaves of control plants and those treated with Si + Fe (Figure 4), which, combined with the reduction in their stomatal conductance relative to optimal growth conditions, contributed to maintaining osmotic balance under conditions of water shortage, and thus demonstrated the adjustment of plants to such stress conditions. The statistically significant difference shown in the increase in ABA content under drought in plants treated with Si + Fe relative to those treated with Si alone, as well as in the case of some other physiological parameters (e.g., osmotic potential or silicon content in individual parts of the plant), suggests that the presence of iron as an additional element next to silicon may play an important role in the plant’s response to drought. The interaction of silicon and iron has been confirmed by studies on the role of Si in alleviating the symptoms of iron deficiency and its distribution in the case of such deficiencies in some plants [36], or the inhibition of iron uptake, e.g., in rice [35]. Analyses focusing on this joint effect on water management parameters in combination with their independent effects may provide the basis for further research in the future.

## 4. Materials and Methods

### 4.1. Plant Materials and Growth under Control Conditions

**Figure 5:**
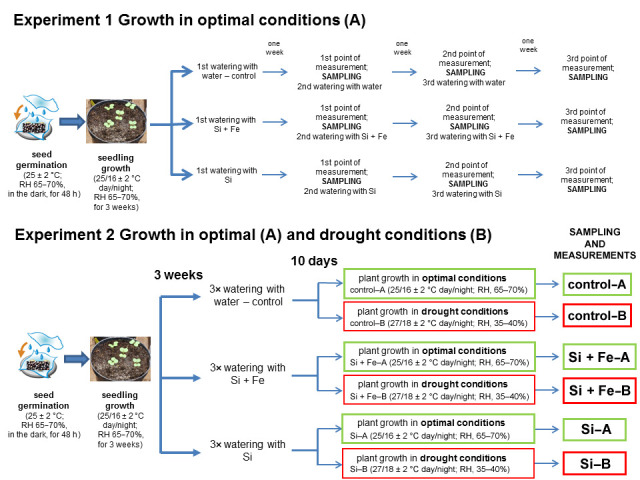
The scheme of two experiments conducted on oilseed rape plants. In both Experiments 1 and 2, plants grew for 44 days under optimal conditions and were watered three times with water (control), silicon and iron (Si + Fe), or silicon (Si). In Experiment 2, the plants then grew for 10 days under optimal conditions (**A**) or drought (**B**).

Seeds of oilseed rape (*Brassica napus* var. *napus* L.) cv. Markus were obtained from the Institute of Plant Protection—National Research Institute, Poznań, Poland, and checked for uniformity. The research encompassed two independent experiments—the first one under optimal water conditions (Experiment 1) and the second one under both optimal water conditions and under drought (Experiment 2). In both cases the initial experimental scheme was the same (Figure 5).

Petri plates were lined with filter paper, on which the seeds were sown. The petri plates with seeds were kept in growth chambers in temperature 25 ± 2 °C, relative humidity (RH) 65–70%, in the dark for 48 h. Next, the seedlings were planted into plastic pots of 1.5 L in volume. Before planting, the pots were tared to equal weight and filled with a homogeneous mixture of garden soil, “black soil” (chernozem) and sand (1:2:1; *v*/*v*), with pH value of 7.0–7.3. Five plants were planted per pot. The plants grew in growth chambers for the first 4 weeks and were then moved to a plastic growth tunnel. They were watered with tap water as needed. Air temperature was 25/16 ± 4 °C day/night, and relative air humidity was fluctuating in the range of 37–80%.

### 4.2. Silicon Treatment

After three weeks of growth, with 2 leaves unfolded—growth stage: leaf development, code 12 on the Biologische Bundesanstalt, Bundessortenamt und CHemische Industrie (BBCH)-scale (canola) [45]—plants were divided into three groups. The first group (control) was watered with 60 mL tap water/1.5 L soil per pot, the second (Si) with 60 mL silicon solution (Orthosilicic acid tetraethyl ester, Sigma-Aldrich, St. Louis, Missouri, USA)/1.5 L soil per pot, and the third (Si + Fe) with 60 mL solution of commercial growth stimulator *Optysil* (Intermag, Olkusz, Poland)/1.5 L soil per pot. The silicon ions content in both solutions was the same at 3.4 mM Si. It should be emphasized that *Optysil* also contains iron (in ethylenediaminetetraacetic acid, EDTA chelate form) in the concentration of 0.00027 mM Fe at the second oxidation stage but it is not enriched with any other ions. Plants were watered three times at weekly intervals. The physiological parameters of plants were always analyzed one week after watering with solutions containing silicon.

### 4.3. Growth under Drought Conditions

After six weeks of growth, with 4 leaves unfolded—growth stage: leaf development, code 14 on the BBCH-scale (canola) [45]—the plants in each of the three groups (control, Si, Si + Fe) were divided into two subgroups (Figure 1). The first group (A) continued under optimal growth conditions, 65–70% of field water capacity (FWC) of the used soil mixture. The second group (B) was subjected to drought for 10 days by limiting water supply and maintaining soil moisture at ca. 30% of FWC. Air temperature was 27/18 ± 2 °C day/night, and relative air humidity was fluctuating in the range of 35–40%. Soil moisture on the day of measurement was 65% of FWC in group A and 30% in group B. Analyses of plants growing under optimal growth conditions and water-deficit were carried out 10 days after the last watering of plants with solutions containing silicon—growth stage: leaf development, code 15 on the BBCH-scale (canola) [45].

### 4.4. Total Silicon Content in Plants

Silicon content analyses were carried out according to the modified method by Masarovic et al. [46]. Above-ground parts (stem and leaves) and underground parts (roots) were cut and then dried in a drying oven at 105 °C for 24 h. The combustion of the samples was carried out in an open mineralizer in borosilicate glass. Three independent samples, each consisting of 10 leaves from 10 different plants, were collected for each treatment. At least three measurements were performed on each sample. 

### 4.5. Leaf Water Content (LWC)

LWC was estimated on the basis of fresh weight (FW) and dry weight (DW) after freeze-drying the samples collected for ABA measurement, according to the formula: LWC = ((LFW − LDW)/LFW) × 100 (%), where LFW—leaf fresh weight, LDW—leaf dry weight. For each of the three independently collected pool samples from one treatment (3 biological replicates), at least three water content measurements (3 technical replicates) were performed, giving in total nine measurements for each treatment.

### 4.6. Osmotic and Water Potential

Water potential (Ψw) and osmotic potential (Ψo) were determined using the dew point method and the HR 33T microvoltmeter (Wescor, Logan, UT, USA). For the measurement of osmotic potential, the collected leaf was pressed in a syringe to completely moisten a filter paper disk (⌀ 5 mm) with cell sap. Then, the disk was placed in the measuring chamber and after 30 min of stabilization, the measurement was performed. When measuring water potential, 3 leaf discs cut using a cork borer were placed in the chamber. The measurement was performed after 40 min of stabilization. The second fully developed leaf (from the top) was selected for both measurements. For each of the 3 independently collected pool samples from one treatment (3 biological replicates), at least 5 technical replicates were performed, giving in total 15 measurements for each treatment.

### 4.7. Estimation of Evapotranspiration

Before the beginning of the drought treatment, the pots with plants were filled up with soil to the same weight. Every day during the drought treatment, the pots were weighed at the same time, and then watered on the weighing scale to the value corresponding to 30% or 65% of FWC for drought and optimal watering treatment, respectively. The differences between pot weights after watering and after 24 h, i.e., before the next watering, were the measure of evapotranspiration—the total amount of water lost from the pot via transpiration and evaporation from pot soil surface. For each of the 3 independently collected pool samples from one treatment (3 biological replicates), at least 5 technical replicates were performed, giving in total 15 measurements (pots) per treatment.

### 4.8. Leaf Stomatal Conductance Measurement

Leaf stomatal conductance (gs) of the fifth oldest leaves of drought-treated and control plants was determined approximately 4 h after the start of the photoperiod using a hand-held AP4 porometer (Delta-T Devices, Cambridge, UK). For each of the three independently collected pool samples from one treatment (3 biological replicates), at least three stomatal conductance measurements (3 technical replicates) were performed, giving in total nine measurements for each treatment.

### 4.9. ABA Level Measurement

Three independent pool samples, each consisting of four leaves coming from two plants, were collected and after FW determination and immediately frozen. Samples were freeze-dried and ground with ball mill MM400 (Retsch, Haan, Germany) in Eppendorf vials, to which 1.5 mL of cold-distilled water was then added. The vials were heated for 3 min in thermoblock set to 90 °C and then shaken overnight at 4 °C in order to extract ABA [47]. The next day, the aqueous extracts were centrifuged for 20 min in a refrigerated centrifuge at 18,000× *g* (MPW-350R, Warsaw, Poland). ABA was measured in the supernatant using the indirect enzyme-linked immunosorbent assay (ELISA) according to Walker-Simmons and Abrams [48]. The antibody used was MAC 252 (Babraham Technix, Cambridge, UK). Absorbance was measured with microplate reader Model 680 (Bio-Rad Laboratories, Hercules, CA, USA) at the wavelength of 405 nm. For each of the three independently collected pool samples from one treatment (3 biological replicates), at least three ELISA measurements (3 technical replicates) were performed, giving in total nine ABA measurements for each treatment.

### 4.10. Statistical Analysis

Statistical analysis was carried out using Statistica v. 13.1 (StatSoft Inc., Dell, Tulsa, OK, USA). Analysis of variance was used to determine the main effects of the treatments on the physiological parameters. Duncan’s multiple range test at the 0.05 probability level was chosen to determine the significance of differences among the treatment means. Additionally, Fisher’s multiple range test at the 0.05 probability level was chosen to determine the significance of differences in evapotranspiration. Two different tests were used because of different variability of the investigated parameters. Correlations between the measured parameters were tested at the probability of *p* < 0.05.

## 5. Conclusions

The presented research shows that despite the widespread belief in poor silicon accumulation by plants of the *Brassicaceae* family, oilseed rape demonstrates a significant increase in the content of this element in the above-ground part of the plant after supplementation with silicon. The observed differences in the silicon accumulation between the individual parts of the leaf—the leaf blade and petiole—may affect their hydraulic conductivity and water balance of the whole plant. The assessment of osmotic adjustment on the basis of selected physiological parameters does not provide unequivocal evidence to conclude that Si actively promotes water accumulation in plants under drought conditions. A slightly better response in the case of some studied physiological parameters, e.g., ABA content, after the application of Si + Fe may suggest that in the case of oilseed rape, an important role is played by the presence of other elements—in this case EDTA chelated iron—which can improve the effectiveness of drought protection. We suggest that the physiological basis of water balance regulation by silicon might be associated with the mechanism of overcoming barriers hindering water uptake by the plant. The indications suggesting the role of this element in improving water uptake by the plant should be the basis for further studies associated with the characteristics of the root system and the biochemistry of chemical components involved in water transport through membranes—including proteins from the aquaporin family—as well as more detailed investigations of the course of the plant’s water transport itself.

## Figures and Tables

**Table 1:** Silicon (Si) content in oilseed rape plants under optimal conditions.

Growth Conditions	Treatment	Shoot (Leaves and Stem) Si (mg/kg)	Leaf Blade Si (mg/kg)	Leaf Petiole Si (mg/kg)
**Optimal conditions**	control	729.8 ^c^	1487.1 ^b^	1182.4 ^b^
	Si + Fe	2159.25 ^a^	1804.3 ^a^	2351.4 ^a^
	Si	1663.35 ^b^	953.1 ^c^	1258.2 ^b^

Plants grew for 44 days under optimal conditions and were watered three times with water (control), silicon and iron (Si + Fe), or only silicon (Si), after which silicon content was measured in the above-ground plant parts (Experiment 1). The means (n = 30) marked with the same letter do not differ significantly (Duncan’s multiple range test, *p* < 0.05).

**Table 2:** Leaf water content and leaf osmotic (Ψo) potential in leaves of oilseed rape.

Points of Measurement	Treatment	Leaf Water Content (%)	Leaf Osmotic Potential Ψo (Mpa)
**One week after first watering**	control	90.09 ^a^	−0.74 ^a^
	Si + Fe	90.10 ^a^	−0.82 ^c^
	Si	89.33 ^a,b^	−0.77 ^a,b^
**One week after second watering**	control	89.47 ^a^	−0.82 ^c^
	Si + Fe	87.87 ^b,c^	−0.83 ^c^
	Si	87.94 ^b,c^	−0.80 ^b,c^
**One week after third watering**	control	89.12 ^a,b^	−0.75 ^a^
	Si + Fe	86.56 ^c^	−0.78 ^b,c^
	Si	87.62 ^c^	−0.75 ^a^

Analysis was performed on leaves of plants growing for 44 days under optimal conditions, collected after each of three weekly waterings with water (control), silicon and iron (Si + Fe), or silicon (Si) (Experiment 1). The means (n = 9 for water content; n = 5 for osmotic potential) marked with the same letter do not differ significantly (Duncan’s multiple range test, *p* < 0.05, applied collectively to all points of measurement).

**Table 3:** Silicon content in oilseed rape plants under drought.

Growth Conditions	Treatment	Shoot (Leaves and Stem) Si (mg/kg)	Leaf Blade Si (mg/kg)	Leaf Petiole Si (mg/kg)
**Drought**	control	889.1 ^c^	1111.5 ^b^	1305.5 ^c^
	Si + Fe	2115.1 ^a^	973.8 ^b^	1731.3 ^b^
	Si	1422.4 ^b^	1548.7 ^a^	3241.5 ^a^

Plants grew first for 44 days under optimal conditions and were watered three times with water (control), silicon and iron (Si + Fe), or only silicon (Si), and afterwards for 10 days under optimal conditions or drought, after which silicon content was measured in above-ground plant parts (Experiment 2). The means (n = 30) marked with the same letter do not differ significantly (Duncan’s multiple range test, *p* < 0.05).

**Table 4:** Leaf osmotic (Ψo) and water (Ψw) potential in leaves of oilseed rape.

Growth Conditions	Treatment	Leaf Osmotic Potential Ψo (MPa)	Leaf Water Potential Ψw (MPa)
**Optimal conditions**	control	−1.13 ^a,b^	−0.86 ^a^
	Si + Fe	−1.08 ^a^	−0.87 ^a^
	Si	−1.20 ^b^	−0.99 ^a^
**Drought conditions**	control	−1.76 ^d^	−1.35 ^b^
	Si + Fe	−1.48 ^c^	−1.30 ^b^
	Si	−1.68 ^d^	−1.46 ^b^

Plants grew first for 44 days under optimal conditions and were watered three times with water (control), silicon and iron (Si + Fe), or only silicon (Si), and afterwards for 10 days under optimal conditions or drought, after which Ψo and Ψw measurements were performed (Experiment 2). The means (n = 5) marked with the same letter do not differ significantly (Duncan’s multiple range test, *p* < 0.05).

## Data Availability

Not applicable.
